# Supplementary material for: The Occurrence of Freshwater Fish-Borne Zoonotic Helminths in Italy and Neighbouring Countries: A Systematic Review
Source: Animals (Basel). 2023 Dec 8;13(24):3793. doi: 10.3390/ani13243793 (PMC10741178; doi:10.3390/ani13243793)
Supplement: Supplementary file 1 [file animals-13-03793-s001.zip › Supplementary file S1.pdf]

WOS core collection	TS=(fish) AND ((((((((((((((((((((TS=(zoonotic parasite)) OR TS=(parasitic zoonosis)) OR TS=(parasitic zoonoses)) OR TS=(cestoda)) OR TS=(cestode)) OR TS=(tapeworm)) OR TS=(Diphyllobothriidea)) OR TS=(Diphyllobothrium)) OR TS=(Dibothriocephalus)) OR TS=(nematoda)) OR TS=(nematode)) OR TS=(roundworm)) OR TS=(Dioctophymatidae)) OR TS=(Eustrongylides)) OR TS=(trematoda)) OR TS=(trematode)) OR TS=(flatworm)) OR TS=(fluke)) OR TS=(Opisthorchiidae)) OR TS=(Opisthorchis)) OR TS=(metorchis)) OR TS=(Clinostomum)) OR TS=(Pseudoamphistomum) AND (((TS=(freshwater)) OR TS=(lake)) OR TS=(river)) OR TS=(internal water)	2.649
Pubmed	((fish[Title/Abstract]) AND ((((((zoonotic parasite[Title/Abstract]) OR (parasitic zoonosis[Title/Abstract])) OR (parasitic zoonoses[Title/Abstract])) OR ((((((cestoda[Title/Abstract]) OR (cestode[Title/Abstract])) OR (tapeworm[Title/Abstract])) OR (diphyllobothriidae[Title/Abstract])) OR (diphyllobothrium[Title/Abstract])) OR (dibothriocephalus[Title/Abstract]))) OR (((((nematoda[Title/Abstract]) OR (nematode[Title/Abstract])) OR (roundworm[Title/Abstract])) OR (Dioctophymatidae[Title/Abstract])) OR (Eustrongylides[Title/Abstract]))) OR (((((((((trematoda[Title/Abstract]) OR (trematode[Title/Abstract])) OR (flatworm[Title/Abstract])) OR (fluke[Title/Abstract])) OR (opisthorchiidae[Title/Abstract])) OR (opisthorchis[Title/Abstract])) OR (metorchis[Title/Abstract])) OR (clinostomum[Title/Abstract])) OR (pseudoamphistomum [Title/Abstract])))) AND ((((freshwater[Title/Abstract]) OR (lake[Title/Abstract])) OR (river[Title/Abstract])) OR (internal water[Title/Abstract]))	1.043
EMBASE	'fish':ti,ab,kw AND ([medline]/lim OR [pubmed-not-medline]/lim) AND ('zoonotic parasite':ti,ab,kw OR 'parasitic zoonosis':ti,ab,kw OR 'parasitic zoonoses':ti,ab,kw OR 'cestode':ti,ab,kw OR 'diphyllobothriidea':ti,ab,kw OR 'diphyllobothrium':ti,ab,kw OR 'dibothriocephalus':ti,ab,kw OR	237

	'nematode':ti,ab,kw OR 'dioctophymatidae':ti,ab,kw OR 'eustrongylides':ti,ab,kw OR 'trematode':ti,ab,kw OR 'flatworm':ti,ab,kw OR 'fluke':ti,ab,kw OR 'opisthorchiidae':ti,ab,kw OR 'opisthorchis':ti,ab,kw OR 'metorchis':ti,ab,kw OR 'clinostomum':ti,ab,kw OR 'pseudoamphistomum':ti,ab,kw) AND ([embase]/lim OR [preprint]/lim) AND ('fresh water':ti,ab,kw OR 'river':ti,ab,kw OR 'lake':ti,ab,kw OR 'internal water':ti,ab,kw) AND ([embase]/lim OR [preprint]/lim)	
